# An empirical study of choosing efficient discriminative seeds for oligonucleotide design

**DOI:** 10.1186/1471-2164-10-S3-S3

**Published:** 2009-12-03

**Authors:** Won-Hyoung Chung, Seong-Bae Park

**Affiliations:** 1 Department of Computer Engineering, Kyungpook National University, Daegu 702-701, South Korea

## Abstract

**Background:**

Oligonucleotide design is known as a time-consuming work in bioinformatics. In order to accelerate and be efficient the oligonucleotide design process, one of widely used approach is the prescreening unreliable regions using a hashing (or seeding) algorithm. Since the seeding algorithm is originally proposed to increase sensitivity for local alignment, the specificity should be considered as well as the sensitivity for the oligonucleotide design problem. However, a measure of evaluating the seeds regarding how adequate and efficient they are in the oligo design is not yet proposed. Here, we propose novel measures of evaluating the seeding algorithms based on the discriminability and the efficiency.

**Results:**

To evaluate the proposed measures, we examine five seeding algorithms in oligonucleotide design. We carried out a series of experiments to compare the seeding algorithms. As the result, the spaced seed is recorded as the most efficient discriminative seed for oligo design. The performance of transition-constrained seed is slightly lower than the spaced seed. Because BLAT seeding algorithm and Vector seeding algorithm give poor scores in specificity and efficiency, we conclude that these algorithms are not adequate to design oligos.

Consequently, we recommend spaced seeds or transition-constrained seeds with 15~18 weight in order to design oligos with the length of 50 mer. The empirical experiments in real biological data reveal that the recommended seeds show consequently good performance. We also propose a software package which enables the users to get the adequate seeds under their own experimental conditions.

**Conclusion:**

Our study is valuable to the two points. One is that our study can be applied to the oligo design programs in order to improve the performance by suggesting the experiment-specific seeds. The other is that our study is useful to improve the performance of the mapping assembly in the field of Next-Generation Sequencing. Our proposed measures are originally designed to be used for oligo design but we expect that our study will be helpful to the other genomic tasks.

## Background

Since the beginning of human genome project, the demand of designing oligonucleotide has been undergoing explosive growth. An oligonucleotide (shortly oligo) is a small DNA sequence (usually ranging from 20 to 70 bp) designed for hybridization only with a targeted position in a target sequence, and the oligonucleotide design is a basic process for many bio-molecular experiments including gene identification, PCR amplification, DNA microarray, and so on. One of the most important issues in oligonucleotide design is to minimize the cross-hybridization event. The usual oligonucleotide designs spend too much time to calculate the hybridization values for all possible oligos and counterparts. Thus, many heuristic algorithms have been applied to this problem as a filter to remove unreliable regions before checking the cross-hybridization. They are clustered into three major categories: multiple alignments [1], suffix tree [2], and hashing algorithm using seeds (shortly seeding algorithm) [3,4]. Among these categories, the seeding algorithm is the most widely used algorithm because of the fast search speed with allowing some mismatches.

The seeding algorithm process consists of a filtering step and an extension step in general. At the filtering step, short fixed-length common words that are found at both query and target sequences are selected. Then at the extension step, it determines whether each word can be extended into a significant alignment. BLAST [3] is the most popular program using this process. BLAST uses fixed-length continuous matches as a template for finding common words, and the template is called a *seed*. Most oligo design programs [5-7] adopt BLAST as a filter. However, the seeding algorithm has a problem of trade-off between sensitivity and search speed. Enlarging the seed size increases the risk of missing true alignments, while shortening it generates more random hits and results in computational slowdown. PatternHunter [4] showed that the problem can be weakened by introducing a non-continuous seed such as "111010010100110111," so-called a *spaced seed*. After the notion of non-continuous seed was presented, the spaced seed has been studied by many researchers in aspects of computational complexity [8-12] as well as adapting the seeds for more specific biological sequences [13,14]. Recently, oligo design programs have been adopting such enhanced seeding algorithms. A oligo design programs ProDesign [15], used YASS [14] to improve its computational speed.

Despite the possibility of speeding up the design time of a seed, a measure of evaluating seeds regarding how adequate and efficient they are in the oligo design has been not yet examined as far as we have explored. We noticed that the seeding algorithms have been developed only to maximize the sensitivity of finding all possible alignments. However, oligonuleotides should be specific to non-target sequences as well as sensitive to the target sequences. Thus, in order to design oligonuleotides for using a seeding algorithm, the seeding algorithm needs to be selected by considering the ability of discriminating target and non-target regions properly.

In this paper, we propose a novel measure of evaluating the seeding algorithms based on the discriminability and the efficiency. By the measure proposed, we examine five seeding algorithms in oligonucleotide design. We carried out a series of experiments to compare the existing seeding algorithms. The results show that the spaced seeding algorithm was generally preferred to the other seeding algorithms. The performance of transition-constrained seeding algorithm was slightly lower than the spaced seeding algorithm. Considering discriminability only, continuous seeding algorithm is as good as the spaced seeding algorithm in the comparison of low weights of the seeds. However, in the others of the comparison, the performance of continuous seeding algorithm degrades rapidly. Because BLAT seeding algorithm and Vector seeding algorithm give poor scores in specificity and efficiency, we conclude that these algorithms are not adequate to design oligos. Consequently, we recommend spaced seeds or transition-constrained seeds with 15~18 weight in order to design oligos with the length of 50 mer. The recommended seeds show consequently good performance in real biological data. We propose a software package, SeedChooser, which enables the users to get the adequate seeds under their own experimental conditions. Our study is valuable to the two points. One is that our study can be applied to the oligo design programs in order to improve the performance by suggesting the experiment-specific seeds. The other is that our study is useful to improve the performance of the mapping assembly in the field of Next-Generation Sequencing. Our proposed measures are originally designed to be used for oligo design but we expect that our study will be helpful to the other genomic tasks.

The rest of the paper is organized as follows. First, we define the performance measures to evaluate seeding algorithms on oligo design: discriminability, efficiency and efficient discriminability. In Result section, the five well-known seeding algorithms are compared with the proposed measures. The five types of the seeds are also estimated with two real biological data sets. We propose a software package which enables to design and evaluate the appropriate seeds with empirical manners. Then we discuss the issues which appeared in the results and draws conclusions. Lastly, we describe how to evaluate a set of the seeds for oligo design in Method section.

## Problem definition

It is a general idea in the oligo design that an ideal seeding algorithm should filter all regions as fast as possible that have no possibility of being chosen as an oligo. However, actually there are three issues to be considered regarding how adequate and efficient a seeding algorithm is in the oligo design. First, a seed should find as many oligos as possible. Second, a seed should not find any non-oligo region. Lastly, a seed should generate hash values as few as possible which are useful to find oligos. There are trade-off relationships among the issues. Therefore, we propose a novel measure of *efficient discriminability *which considers all of them. This measure is based on the two metrics: *discriminability *and *efficiency*. The discriminability is a balance between sensitivity and specificity to minimize both false positives and false negatives. The efficiency is the proportion of useful regions filtered by the seeding algorithm.

### Discriminability

**Figure 1:**
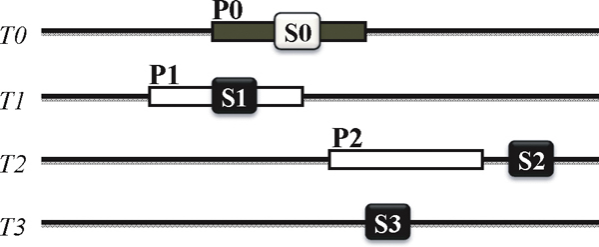
**The illustration of the effect of seeding on oligo design**. The oligos are selected from target sequences using a seed. *T*1, *T*2 and *T*3 are the target sequences. *P*1 and *P*2 are the matched oligos for an oligo *P*0, while *S*1, *S*2 and *S*3 are the matched hashes for *S*0 by a seed.

The illustration of the effect of a seeding is shown in Figure 1. It shows the oligo selection in the event of filtration using a seeding algorithm. Let the hash value filtered by a seed be a 'seed hash'. For an oligo *P*0, the seed hash S0 in P0 is used to find possible oligos in the target sequences. When a seed hash *S*1 is occurred in the oligo *P*1. it is called as 'hit'. This is a desirable case because the seeding finds an oligo successfully. It is also possible that the seed hashes *S*2 and *S*3 fail in finding their oligos. Another undesirable case is that oligo *P*2 does not have any seed hash. These cases are summarized as follows:

• True positive (TP): the seeding hits oligos (*S*_*O*_), or oligos contains at least a seed hash (*O*_*S*_).

• False negative (FN): oligos have no seed hash ().

• False positive (FP): the seeding misses oligos ()

• True negative (TN): the seeding does not hit any non-oligo region.

The sensitivity and the specificity of a seeding in the oligo design are the most common and widely-used measures. However, they have a problem that the amount of true negatives is not considered. Therefore, instead of them, it is more appropriate to use precision and recall in evaluating a seeding in the oligo design. They have ability to measure false positives and false negatives. In addition, they can be merged into one easily. Precision *P *is defined as the proportion of seed hashes hitting oligos to all seed hashes, while recall *R *is the proportion of oligos containing the matched seed hashes to all selected oligos.

For the unified measure *discriminability*, F-measure is used which is the weighted harmonic mean of precision and recall. In real oligo design problem, it is needed to set the different weight between precision and recall. Recall is more valuable than precision in minimizing false negatives, while precision is more valuable than recall in minimizing false positives. This is controlled by importing a non-negative parameter *α *into the F-measure. Therefore, the discriminability, *F*_*α *_is given as

Then, a discriminative seed is defined as a seed that has the maximum discriminability. The discriminability *F*_*α *_has following properties: The maximum value of the discriminability is 1 and it can be obtained only when both *P *and *R *are 1. When the discriminability has the maximum value, there is no false positive and no false negative. The balance of the sensitivity and the specificity is controlled by *α*. Increasing *α *over 1, the weight of precision becomes higher than that of recall. It makes *F*_*α *_sensitive to false positives. Decreasing *α *below 1, the weight of precision becomes lower than that of recall. It makes *F*_*α *_sensitive to false negatives.

### Efficiency

The efficiency of a seed on oligo design can be measured by two points: (i) the duplicated generation of hash values and (ii) the average number of seed hashes in an oligo. Some seeding algorithms allowing some mismatches such as BLAT [16] and VectorSeeds [13] generate multiple hash values at a single position. It increase sensitivity in that it generates more hash values than simple seeding algorithms, in most cases, it over-generates hash values to find an oligo. Therefore, it is desirable to minimize the duplicated hash values during the generation time. The duplication rate of the generated seed hashes, *D *is defined as follows.

Another consideration for the efficiency is about the number of seed hashes in an oligo. Since the length of an oligo is longer than that of a seed, an oligo could be found by multiple seed hashes. However, only one seed hash is sufficient in finding an oligo. The average rate of seed hashes in an oligo, *A *is defined as follows.

Both the duplication rate *D *and the average rate *A *are at least 1 because the value of numerator includes the value of denominator. Each of the rates is normalized as follows: 1/(1 + *weight*·log *rate*). The efficiency is defined as the multiplication of the normalized *D *and *A*. The weight *β *for *D *and the weight *γ *for *A *are both ranged from 0 to 1. Then the efficiency *E*_*β*, *γ *_is given as

Since the values *D *and *A *are non-negative and their minimum values are 1, the maximum value of the efficiency *E*_*β*, *γ *_is 1. It means that an oligo contains only one seed hash when *E*_*β*, *γ *_has the maximum efficiency, 1.

### Efficient discriminability

Finally, we define the *efficient discriminability*, *G*_*α*, *β*, *γ *_as a product the discriminability (*F*_*α*_) and the efficiency (*E*_*β*, *γ*_).

Then, the efficient discriminative seed is the seed that has the maximum efficient discriminability value for given *α*, *β *and *γ*. When all the parameters *α*, *β *and *γ *are not zero, the seed with the maximum value of *G*_*α*, *β*, *γ *_is optimal. The value of *G*_*α*, *β*, *γ *_is maximized when both *F*_*α *_and *E*_*β*, *γ *_are maximized. According to the definitions of *discriminability *and *efficiency*, the optimal seed has no false positive and false negative, and it appears only in one oligo.

## Results

We compared the performance of the five seeding algorithms (continuous, spaced, transition-constrained, BLAT, and Vector) on oligo design in perspective. The brief descriptions of those seeding algorithms are found at 'Seeds for Assessment' in Method. In order to estimate the performance of the seeding algorithms, they were evaluated by three measures, discriminability, efficiency, and efficient discriminability, respectively. The weight parameters *α*, *β*, and *γ *were set to 1 by default.

Empirically the selected seeds which are believed to represent their seeding algorithms were estimated by the measures and plotted by the weight of the seeds. The reason why the seeds are plotted by weight is that the seeds having the same weight are generally considered to spend the same computing costs. We selected 85 seeds for test empirically as shown in Table S1 and S2 of the Additional File 1. Nineteen seeds were selected respectively for continuous seeding algorithm, spaced seeding algorithm, and trnasition-constrained seeding algorithm granting different weights from 7 to 25. For BLAT seeding algorithm and Vector seeding algorithm, fourteen seeds which have the different weights from 14 to 27 were selected, respectively. The above two seeding algorithms allowing mismatches in their seeds are exceedingly more sensitive than the other seeding algorithms. Generally, a seed's weight is in inverse proportion to sensitivity. That is, if a seed's weight is increased, the seed's sensitivity is decreased. So We skipped the selection of the seeds below 14-weight instead of the additional selection of 26 and 27-weight seeds.

We tested the selected seed on a set of the simulated data and the two sets of biological data. The simulated data is a set of artificially generated oligos and target sequences. The biological data are obtained from an oligo design program HPD [17]. The details of the data are described at 'Sequences for Assessment' in Method. To summarize the experimental results, we identified that the 16-weight spaced seed showed the highest performance among the examined seeds in accordance with the efficient discriminability. Without the considering efficiency, the 12-weight spaced seed achieved the highest performance. The results show that spaced seeding algorithm is generally preferred to the other seeding algorithms in the viewpoint of the efficient discriminability. The results of transition-constrained seeding algorithm are as good as the results of the spaced seeding algorithm. Considering discriminability only, continuous seeding algorithm is as good as the spaced seeding algorithm in the comparison of low weights of the seeds. However, in the others of the comparison, the performance of continuous seeding algorithm degrades rapidly. We also identify that the seeding algorithms which allow mismatches in the seeds show high performances only considering sensitivity. Therefore, both BLAT seeding algorithm and Vector seeding algorithm are not adequate to design oligos. The recommended seeds show consequently good performance in real biological data.

### Discriminability of the five seeding algorithms

**Figure 2:**
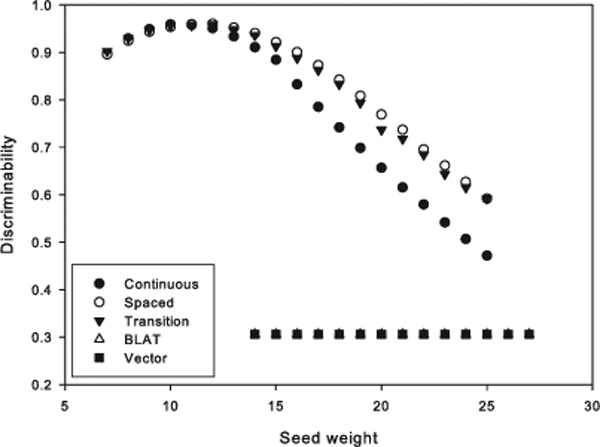
**The discriminability of the five seeding algorithms**.

Under the default parameter (*α *= 1), the comparison of the five seeding algorithms in considering of the discriminability is given in Figure 2. The X-axis of this figure is the weight of seed, and the Y-axis is the value of discriminability. The *weight *implies the real size of a seed to be compared. In the figure, the 12-weight spaced seed ranks the highest where its discriminability is 0.96 at highest peak point. The 11-weight continuous seed and the 12-weight transition-constrained seed also achieve the next highest results. Their discriminabilities are as high as the best score of the spaced seed. These two seeding algorithms show similar curves, where the curve of the spaced seed is slightly above that of the continuous seed. On the other hand, all BLAT seeds and all Vector seeds show very low discriminability results around 0.3. Their low discriminabilities come from their extreme sensitivity. Their precisions are both equal to 1, while the recall is just 0.18 for both algorithms.

### Efficiency of the five seeding algorithms

**Figure 3:**
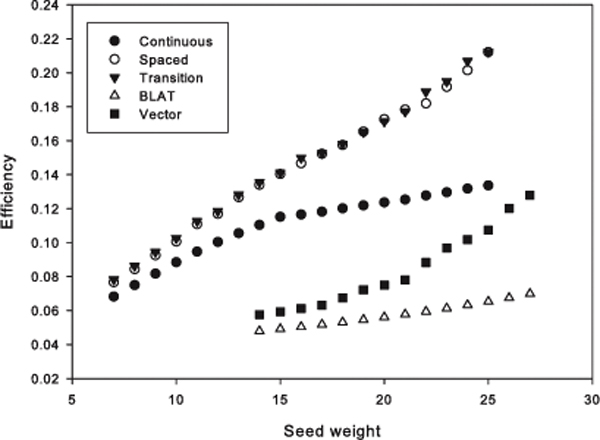
**The efficiency of the five seeding algorithms**.

The efficiency monotonously increases with the weight of a seed increased (see Figure 3). The spaced seed and the transition-constrained seed show the best efficiency. The curve of the continuous seed is lowered after that of the 15-weight of the seed while the curve of Vector seed increases steadily.

### Efficient discriminability of the five seeding algorithms

**Figure 4:**
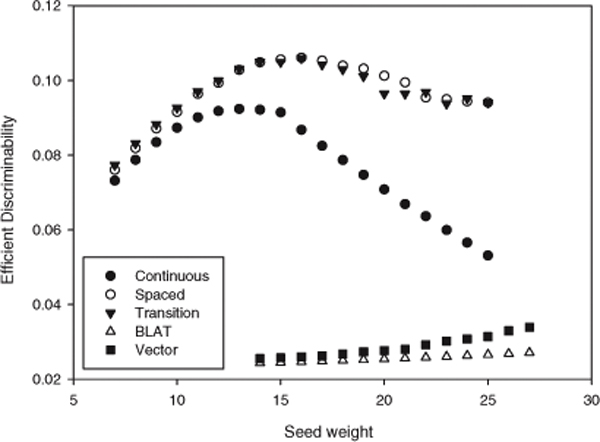
**The efficient discriminability of the five seeding algorithms**.

The efficient discriminabilities of the five seeds were compared with the parameters of *α*, *β *and *γ *fixed as the default value. Figure 4 shows the comparison results. In this figure, the Y-axis is the efficient discriminability. The highest efficient discriminative seed is the 16-weight spaced seed of which efficient discriminability was 0.133703. All spaced seeds were positioned at the first or second ranks. The transition-constrained seeds showed similar curve with the spaced seeds, but the highest result was lower than that of the spaced seed. The efficient discriminabilities of the continuous seeds declined steeply above the weight 15. all BLAT seeds and all Vector seeds were ranked the lowest expectiviely.

### Seed estimation with the biological data sets

Performance of the seeds is estimated with the two biological data sets, *pmoA *and *nirS*. The results are listed in Table 1 and 2. From *pmoA *data we identified 19-weight spaced seed as the highest efficent discriminative seed. The estimated value is 0.2323. The highest discriminative seed was 18-weight transition-constrained seed scored with 0.8857. For *nirS *data, the best efficient discriminative seed was 14-weight spaced seed scored with 0.1697. This seed was also the best discriminative seed for the data set. We noticed that the estimation results of *pmoA *data set followed the simulation results. The results of *nirS *data set was lower than the simulation results. We predict that it was related to the average similarity of the data set. Therefore we conclude that longer spaced seed is good to the data set showing higher similarity and shorter spaced seed is good to the diversely distributed data set.

**Table 1:** Evaluation results for pmoA data set

	Efficient Discriminability			Discriminability			Efficiency		
Weight	Cont	Spaced	Trans	Cont	Spaced	Trans	Cont	Spaced	Trans
7	0.09071	0.1027	0.1025	0.5341	0.5826	0.5848	0.06188	0.07246	0.07246
8	0.1067	0.1184	0.1167	0.6011	0.6443	0.6382	0.07627	0.08734	0.08568
9	0.122	0.1318	0.1295	0.659	0.6806	0.6728	0.09095	0.0999	0.09755
10	0.1335	0.1437	0.1439	0.6949	0.7161	0.7189	0.1023	0.112	0.1124
11	0.1447	0.1517	0.1532	0.7245	0.7317	0.7378	0.1135	0.1196	0.1214
12	0.1538	0.1611	0.1561	0.7447	0.756	0.738	0.1245	0.1295	0.1244
13	0.1638	0.1788	0.1752	0.7657	0.7997	0.7893	0.135	0.1503	0.146
14	0.174	0.1845	0.1875	0.7839	0.8129	0.8186	0.146	0.1591	0.1606
15	0.1597	0.2016	0.2016	0.7323	0.8374	0.8343	0.1496	0.1797	0.1791
16	0.1633	0.2043	0.2045	0.7356	0.8383	0.8392	0.1584	0.1887	0.1879
17	0.1679	0.2187	0.2161	0.7412	0.8697	0.8605	0.1676	0.2046	0.1998
18	0.1561	0.2259	0.229	0.6971	0.878	0.8857	0.1713	0.2144	0.2125
19	0.1562	0.2323	0.2269	0.6895	0.8697	0.8546	0.1794	0.2285	0.221
20	0.1622	0.2134	0.2148	0.6977	0.796	0.8044	0.1892	0.2349	0.2308
21	0.1575	0.2249	0.2223	0.6741	0.8119	0.8099	0.1955	0.2494	0.2444
22	0.1411	0.2085	0.208	0.6153	0.7535	0.7527	0.1976	0.2514	0.2486
23	0.1414	0.1998	0.2004	0.6087	0.7056	0.7085	0.2071	0.259	0.2616
24	0.1421	0.2209	0.2168	0.6028	0.7285	0.7119	0.2163	0.2855	0.2936
25	0.1318	0.2313	0.2216	0.5627	0.7386	0.7069	0.2188	0.3029	0.2995

*Cont *indicates the continuous seed type, *Spaced *indicates the spaced seed type, and *Trans *indicates the transition-constrained seed type.

**Table 2:** Evaluation results for nirS data set

	Efficient Discriminability			Discriminability			Efficiency		
Weight	Cont	Spaced	Trans	Cont	Spaced	Trans	Cont	Spaced	Trans
7	0.0493	0.05717	0.05845	0.2952	0.3239	0.3327	0.02892	0.03411	0.03505
8	0.07998	0.08818	0.08992	0.4637	0.4877	0.499	0.05206	0.05835	0.05991
9	0.1073	0.1186	0.1191	0.6056	0.6374	0.6399	0.07727	0.08782	0.08781
10	0.1263	0.1425	0.1415	0.6991	0.7474	0.7443	0.09885	0.1155	0.1147
11	0.1406	0.1506	0.1528	0.7632	0.7766	0.7884	0.1175	0.1275	0.1315
12	0.1425	0.1558	0.1538	0.7793	0.8008	0.7941	0.1329	0.1379	0.1364
13	0.1438	0.1657	0.1657	0.7866	0.8397	0.8396	0.1449	0.1607	0.1597
14	0.1429	0.1697	0.1629	0.7833	0.8517	0.8278	0.1549	0.1712	0.1691
15	0.1401	0.1627	0.1659	0.7702	0.8193	0.8306	0.163	0.1807	0.182
16	0.138	0.1608	0.1637	0.7581	0.8132	0.8231	0.1687	0.185	0.186
17	0.138	0.1631	0.1647	0.7533	0.8148	0.8216	0.1734	0.1902	0.1913
18	0.1315	0.1622	0.1643	0.7224	0.806	0.8131	0.1754	0.193	0.1932
19	0.1299	0.1634	0.1639	0.711	0.7965	0.7985	0.178	0.1991	0.1987
20	0.1293	0.1513	0.1513	0.7037	0.7414	0.7419	0.1808	0.2003	0.2006
21	0.129	0.1536	0.1578	0.6972	0.7428	0.7569	0.1833	0.2041	0.2048
22	0.1284	0.1487	0.1491	0.6894	0.7169	0.719	0.185	0.2054	0.2057
23	0.1295	0.1504	0.151	0.6883	0.7014	0.7033	0.1873	0.2133	0.2136
24	0.1274	0.1538	0.1591	0.6747	0.6959	0.7036	0.1883	0.2193	0.2253
25	0.128	0.1496	0.1533	0.6714	0.6716	0.6727	0.1902	0.2224	0.2268

*Cont *indicates the continuous seed type, *Spaced *indicates the spaced seed type, and *Trans *indicates the transition-constrained seed type.

### SeedChooser: seed evaluation and recommendation tools

The results of the above empirical test yield clues to the guideline of selecting an appropriate seed on considering discriminability as well as efficiency. Based on the results, the users may predict which seeding algorithm is prefer to their tasks. However, they really want to know the most appropriate seed length and weight as well as the adequate seeding algorithm in detail. To maximize the effect of the seed recommendation in practice, we constructed the software package including the evaluation process and design process.

We built a user-friendly package of the tools to provide both seed evaluation and seed recommendation. It consists of three programs; SeedChooser, SeedEvaluator, and OligoGenerator. SeedChooser is the main program which recommends a good seed by three parameters *α*, *β *and *γ *SeedEvaluator is the program which evaluates a set of the input seeds by the parameters. OligoGenerator is the program to generate a set of oligos for the desired experimental conditions.

## Discussion

### Multiple seed selection method is not good at aligo design

The seeding algorithms wihch allow some mismatches in the seeds, the BLAT seeding algorithm and the Vector seeding algorithm, is originally proposed to increase sensitivity intentionally by generating multiple seed variations from a seed. These algorithms have been successfully applied to the specific-purpose alignments which are required very high sensitivity. Protein sequence alignment is a good example of the algorithms. However, this is not efficient in the oligo design. In the experiments of the discriminability, recalls are always 1 with the all possible weights, but the precisions are as low as 0.18. It implies that BLAT seed and Vector seed find all oligos since they are too sensitive, but too many seeds are found in non-oligo regions. Thus, they show lower discriminability than other seeding algorithms. The multiple selection of seeds results in also low efficiency. This is because too many seeds are found to get a single oligo. Therefore, they are neither discriminative nor efficient in the oligo design.

### The effect of the weight parameters

Generally, precision is in proportion to the seed weight and recall in reverse proportion to the seed weight. Since the discriminability is the harmonic mean of precision and recall, the highest discriminative seed is found at the cross-point of the precision curve and the recall curve with given one to all weight parameters. (see Figure S1 in the Additional File 2) The parameter *α *which is the weight parameter controlling the balance of precision and recall forces the user's intention. As increasing the parameter *α*, discriminability gets more dependent to the precision. (see Figure S2 in the Additional File 2) While discriminability gets more dependent to the recall as decreasing *α*. A user should choose the lower weighted seeds or the sensitive seeds in order to do the lossless filtration. The best discriminability of *α *= 2^-8 ^is 0.998985, and that of *α *= 2^8 ^is 0.999119, whereas that of *α *= 1 is 0.959362. We also noticed that the discriminability changes in proportion to the seed weight even if precision and recall are fixed. Therefore, the value of discriminability should be compared with between the seeds which have the same weight. The weights of *β *and *γ *for efficiency should be also considered in the same way. Figure S3 in the Additional File 2 is the graphical view of the effect of *β *and *γ *with given the rates fore efficiency *D *= 0.28 and *A *= 0.35.

### An efficient discriminative seed improves the oligo design performance

The oligo design process using a seeding algorithm consists of two steps. The first step is a fast filtration of the unreliable regions for all possible oligos using a seeding algorithm. Since a seeding algorithm uses a hashing data structure, the filtration by the seeding can be executed fast in the linear time. The second step is an accurate filtration step by simulating hybridization. The time complexity of this step is generally quadratic. For example, the 11-weight BLAST seed saves 10% of the seed hashes compared with the 7-weight blast seed with the cost of 1.8% missing of the true positives. It reduces the computational time of the second step up to 81%. The first step also saves the computational time by the amount of 10%. Therefore, the selection of the efficient discriminative seeds reduces the cost of the oligo design by speeding up the computational time with the little loss of accuracy.

### Seed evaluation for next-generation sequencing

Recently, introduction of the new strategies for high-throughput DNA sequencing dramatically reduced the cost of genome sequencing. However, the great sequencing performance of these new technologies is come at the expense of the considerable shorten of read lengths. For example, a typical run of the Illumina Genome Analyzer yields about 50 million reads. But the read size is only 32~40 [18]. One of the promising applications is the re-sequencing projects among the applications of the Next-Generation Sequencing. The object of the re-sequencing project is to reconstruct a sample genome and find genomic variations by mapping the reads to a reference genome. The mapping process raises two new computationally challenging problems. One is that the vast amount of the data requires much faster mapping speed. The other is the mapping of the error-containing reads to the correct positions of the reference genome. Most previous works for mapping process have been used the indexing strategy in order to solve the problems [19]. The representative indexing strategy is to construct indices based on exact matches of length *k *(*k*-mer). The reads sharing a *k*-mer are only compared with each other.

The notable point of *k*-mer indexing strategy is that it is exactly the same process of the first step of the oligo design. The *k*-mer is directly regarded as a continuous seed because both are the templates of the exact matches. Our evaluation measure of the seeds can be used for improving the performance of the mapping assembly due to the following reasons. First, efficiency calculates the expected cost of constructing the *k*-mer indices. Second, discriminability calculates the compromisable point of sensitivity and specificity. Finally, it is allowed to incorporate the five well-known seeding algorithms while selecting a best seed for the mapping assembly. Discontinuous seeds including spaced seed will improve the mapping sensitivity without loss of the specificity in mapping assembly. Recently, Lin et al. [20] pointed out those problems and proposed a mapping assembly tool as a solution by introducing the spaced seed. Lin et al. described a disadvantage of the exact matching process and proved that the spaced seeding can achieve full mapping sensitivity. We ensure that our proposed measures and the developed software will be contributed to the Next-Generation Sequencing field.

## Conclusion

In this paper, we proposed a novel measure of evaluating the seeding algorithms based on the discriminability and the efficiency. By the measure proposed, we examined five well-known seeding algorithms: continuous, spaced, transition-constrained, BLAT, and Vector. From the results, we concluded the comparison of the seeds as below. The spaced seeding algorithm was generally preferred to the other seeding algorithms. The performance of transition-constrained seeding algorithm was slightly lower than the spaced seeding algorithm. The BLAT seeding algorithm and Vector seeding algorithm were not adequate to design oligos because the poor scores in specificity and efficiency. Consequently, we recommend spaced seeds or transition-constrained seeds with 15~18 weight in order to design oligos with the length of 50 mer. The recommended seeds showed consequently good performance in real biological data.

We tested the effect of three weight parameters for discriminability and the efficiency. The highest discriminative seed was found at the cross-point of the precision curve and the recall curve with given one to all weight parameters. Performance of the seeds was estimated with the two biological data, *pmoA *and *nirS*. The estimation of the real data showed that the longer spaced seed was good to the data having higher similarities in their alignments and shorter spaced seed was good to the diversely distributed data. We also proposed a user-friendly package of the tools to provide both seed evaluation and seed recommendation, which enables the users to get the adequate seeds under their own experimental conditions.

We conclude this paper after pointing two promising usages. One is that our study can be applied to the oligo design programs in order to improve the performance by suggesting the experiment-specific seeds because this work is originally designed to elevate the performance of the existing programs. The other is that the measures proposed by here can be extended to the general purpose to evaluate and recommend the seed-like instances. Therefore it can be applied to any kind of studies such as the mapping process in the Next-Generation Sequencing as well as the oligo design and the sequence alignment problems.

## Methods

An overview of our experiments is given as follows.

1. A set of sequences is prepared. The sequences which are randomly generated are used for this work.

2. A set of all possible oligos and their counterparts are created from the prepared sequences. The oligo design criteria and selection process are described below.

3. Three parameters of *α*, *β *and *γ *of *G*_*α*, *β*, *γ *_are assigned according to the conditions of the oligo design.

4. A seed to be examined is selected. This work examines five seeding algorithms by changing seed weights.

5. All possible seed hashes generated from the prepared sequences are stored in a hash data structure. The hash key is a string filtered by the given seed, and the hash values are the sequence indexes and positions where each hash is found. The number of generated hashes is stored to calculate the duplication rate.

6. The discriminability and the efficiency are computed from the results of step 2 and 4. Here, this step produces the value of the efficient discriminability *G*_*α*, *β*, *γ *_for the seed selected at step 3.

7. Repeat from step 4 to step 6 with a query seed changed. The results are sorted and the best efficient discriminative seed is informed.

### Sequences for assessment

#### Simulated data set

We prepare a set of randomly generated sequences. The set consists of 100 artificial sequences with size of 50 bp generated by Bernoulli alignment model. Each sequence is mutated with 5,000 variations by imposing mismatch.

#### Biological data set

Two biological data sets *pmoA *and *nirS *are obtained from the example sequences of HPD [17]. They are ecologically important genes involved in the nitrogen and carbon cycles: nitrite reductase (*nirS*) and methane monooxygenase (*pmoA*). A sample set that contains 47 *nirS *sequences having 64% of average identity was selected from 421 *nirS *sequences. Another sample set that contains 50 *pmoA *sequences having 85% of average identity was picked from 490 *pmoA *sequences.

### Oligo selection

A set of all possible oligos is created based on the oligo size from the sequences. The next step is to find all counterparts for each oligo. Since finding counterparts from all sequences is time-consuming, the possible counterparts are obtained by FASTA [21] which is a slow but very sensitive local alignment tool. The oligo and its counterpart are aligned using CLUSTALW [22]. The identity and continuous match are calculated from the alignment. Finally, the free energy is obtained using OligoArrayAux [23]. According to the guideline for oligo design described below, all target positions are classified as a hybridizable one or not. This step produces a set of all possible oligos and their hybridizable counterparts.

### Oligo design criteria

The oligo design criteria are related to a bio-chemical process, hybridization. The first study of the hybridization criteria [24] suggested two measures for 50 bp oligo: sequence identity and continuous match length. Recent study [25] added a free energy threshold: over 85% identity, over 15 bp continuous matches, and lower -30 kcal/mol in free energy. In our study, the free energy threshold is set to be -40 kcal/mol by using OligoArrayAux, the program based on Zuker's free energy model [26] instead of He's model.

According to our simulation, the free energy threshold provided by He's model is inferior to -40 kcal/mol threshold with Zuker's model. The oligo and target position hybridize each other when at least one of three criteria is over its threshold.

### Seeds for assessment

Five seeding algorithms which have been proposed for local alignment are examined.

#### Continuous seed

it uses a hashing approach to find all matching *k*-tuples. A 11-bp-length seed ("11111111111") is used at BLAST, and a 28-bp-length seed is used at MegaBlast [27].

#### Spaced seed

PatternHunter [4] uses *k *non-consecutive letters as a seed. Due to the relative positions of the *k *letters, it is called a spaced seed model (or simply, a spaced seed). A 18-bp-length seed containing 11-bp matches ("101101100111001011") is used at PatternHunter.

#### Transition-constrained seed

A transition-constrained seed [14] consists of the ternary alphabet 1, @, 0, where @ stands for a match or a transition mismatch (A ↔ G, C ↔ T). This seed is a variation of the spaced seed including transition related states ("1110@10010@1010111").

#### Blat seed

BLAT is a continuous seed allowing one or two mismatches at any positions of the seed.

#### Vector seed

A Vector seed is a generalized seed by combining the idea of BLAT seed and spaced seed. Since each position of the seed has a position-specific weight, the seed looks like a sequence of numbers. Thus, it is called as a 'Vector' seed (For instance, "12022012000012").

## Availability and requirements

**Project name**: SeedChooser

**Project home page**: http://ml.knu.ac.kr/~whchung/seedchooser.html

**Operating system(s)**: Windows XP and above, Linux

**Programming languages**: Python

**Other requirements**: Python version 2.3 or above, CLUSTALW (available at http://www.ebi.ac.uk/Tools/clustalw/index.html) and UNAFOLD (available at http://dinamelt.bioinfo.rpi.edu/download.php) for OligoGenerator.

**License**: The SeedChooser software is provided "as is" with no guarantee or warranty of any kind. SeedChooser is freely redistributable in binary format for all non-commercial use. Source code is available to non-commercial users by request of the primary author. Any other use of the software requires special permission from the primary author.

**Any restrictions to use by non-academics**: None

## Competing interests

The authors declare that they have no competing interests.

## Authors' contributions

Chung conceived the new idea and carried out model building and empirical analysis. Park initiated, supervised and coordinated the project. All authors wrote the manuscript and approved the final version.

## Note

Other papers from the meeting have been published as part of *BMC Bioinformatics* Volume 10 Supplement 15, 2009: Eighth International Conference on Bioinformatics (InCoB2009): Bioinformatics, available online at http://www.biomedcentral.com/1471-2105/10?issue=S15.

## Supplementary Material

Additional file 1 List of the seeds used in the experiment: continuous seeds, spaced seeds, and transition-constrained seeds (19 instances, respectively) BLAT seeds and Vector seeds (14 instances, respectively). Click here for file

Additional file 2 Figures for the effect of the weight parameters: Figure S1 - Relation of precision, recall and discriminability, Figure S2 - Discriminability according to values of *α*, and Figure S3 - Efficiency according to values of *β *and *γ*. Click here for file
