# Supplementary material for: An empirical study of choosing efficient discriminative seeds for oligonucleotide design
Source: BMC Genomics. 2009 Dec 3;10(Suppl 3):S3. doi: 10.1186/1471-2164-10-S3-S3 (PMC2788383; doi:10.1186/1471-2164-10-S3-S3)
Supplement: Additional file 1 — List of the seeds used in the experiment: continuous seeds, spaced seeds, and transition-constrained seeds (19 instances, respectively) BLAT seeds and Vector seeds (14 instances, respectively). [file 1471-2164-10-S3-S3-S1.pdf]

Additional file 1 for "An empirical study of choosing efficient discriminative seeds for oligonucleotide design"

Seeds used in the experiments

Table S1: Seeds used in the experiment (part 1/2). Type *B* indicates the continuous seed and type *S* indicates the spaced seed. Column *length* represents the length of a seed and column *weight* represents the number of '1' in a seed. In a seed, '1' means matching position and '0' means don't care position.

type	length	weight	seed
B	7	7	1111111
B	8	8	11111111
B	9	9	111111111
B	10	10	1111111111
B	11	11	11111111111
B	12	12	111111111111
B	13	13	1111111111111
B	14	14	11111111111111
B	15	15	111111111111111
B	16	16	1111111111111111
B	17	17	11111111111111111
B	18	18	111111111111111111
B	19	19	1111111111111111111
B	20	20	11111111111111111111
B	21	21	111111111111111111111
B	22	22	1111111111111111111111
B	23	23	11111111111111111111111
B	24	24	111111111111111111111111
B	25	25	1111111111111111111111111
S	13	7	1011010100011
S	14	8	11011010100011
S	15	9	110110101000111
S	16	10	1101100011010111
S	18	11	111010010100110111
S	18	12	111010110100110111
S	20	13	11101011001100101111
S	21	14	111011100101100101111
S	23	15	11110010101011001101111
S	23	16	11110110011010101101111
S	24	17	111101010111001101101111
S	25	18	1111011001110101011011111
S	27	19	111101010111001101101101111
S	29	20	11101100101110101011011111011
S	30	21	111101101001110101011011111011
S	31	22	1111001111110100110111011011011
S	34	23	1110110001100111101111011001111011
S	36	24	111100111001001110001111101101111011
S	37	25	1111001110011111000010101111101111011

Table S2: Seeds used in the experiment (part 2/2). Type *T* indicates the transition-constrained seed, type *L* indicates the BLAT seed. and type *V* indicates the vector seed. Column *length* represents the length of a seed. The weight of a transition-constrained seed is calculated as the number of '1's plus the half number of '@'s. The weight of BLAT and Vector seed is given as one less than the number of '1's in the seed. It implies that the seed is allowed one mismatch at any position marked with '1'. In a seed, '1' means matching position, '0' means don't care position and '@' means transition matching (A-G or C-T) position.

type	length	weight	seed
T	13	7	101@0101@0011
T	14	8	1101@0101@0011
T	15	9	1101@0101@00111
T	16	10	1101100@110101@1
T	18	11	11101@0101001@0111
T	18	12	11@01011010011@111
T	20	13	11@01@11001100101111
T	21	14	11101@1001011@0101111
T	23	15	1111001@10101100@101111
T	23	16	111@0110011@10101101111
T	24	17	1@11010101110011011@1111
T	25	18	1111011001@10101@11011111
T	27	19	111@01010111@01101101101111
T	29	20	1@1011@01011@0101011@11111011
T	30	21	111@0110100111@101011011111011
T	31	22	111100111@1101001101110111@11011
T	34	23	1110110@0110011@101111011001111011
T	37	24	1111@01110010011@0001@11110@101111011
T	38	25	1111001@11001111100001@001111101111011
L	15	14	111111111111111
L	16	15	1111111111111111
L	17	16	11111111111111111
L	18	17	111111111111111111
L	19	18	1111111111111111111
L	20	19	11111111111111111111
L	21	20	111111111111111111111
L	22	21	1111111111111111111111
L	23	22	11111111111111111111111
L	24	23	111111111111111111111111
L	25	24	1111111111111111111111111
L	26	25	11111111111111111111111111
L	27	26	111111111111111111111111111
L	28	27	1111111111111111111111111111
V	23	14	11110010101011001101111
V	23	15	11110110011010101101111
V	24	16	111101010111001101101111
V	25	17	1111011001110101011011111
V	27	18	111101010111001101101101111
V	29	19	11101100101110101011011111011
V	30	20	111101101001110101011011111011
V	31	21	1111001111110100110111011011011
V	34	22	1110110001100111101111011001111011
V	36	23	111100111001001110001111101101111011
V	37	24	1111001110011111000010101111101111011
V	38	25	11101100011100111100111110110011111011
V	40	26	1111010111001100111000111011011101111011
V	41	27	11110101110011111100001100101111101111011
