# Supplementary material for: An empirical study of choosing efficient discriminative seeds for oligonucleotide design
Source: BMC Genomics. 2009 Dec 3;10(Suppl 3):S3. doi: 10.1186/1471-2164-10-S3-S3 (PMC2788383; doi:10.1186/1471-2164-10-S3-S3)
Supplement: Additional file 2 — Figures for the effect of the weight parameters: Figure S1 - Relation of precision, recall and discriminability, Figure S2 - Discriminability according to values of α, and Figure S3 - Efficiency according to values of β and γ. [file 1471-2164-10-S3-S3-S2.pdf]

Additional file 2 for "An empirical study of choosing efficient discriminative seeds for oligonucleotide design"

Figures for the effect of the weight parameters

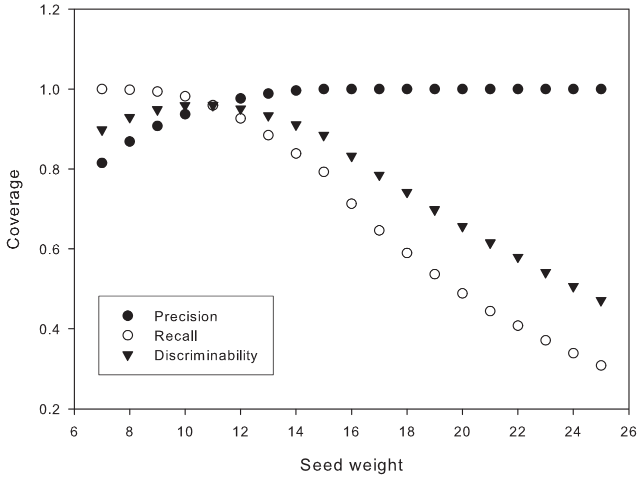

Figure S1: Relation of precision, recall and discriminability. The coverage score of discriminability for each seed was plotted by its weight.

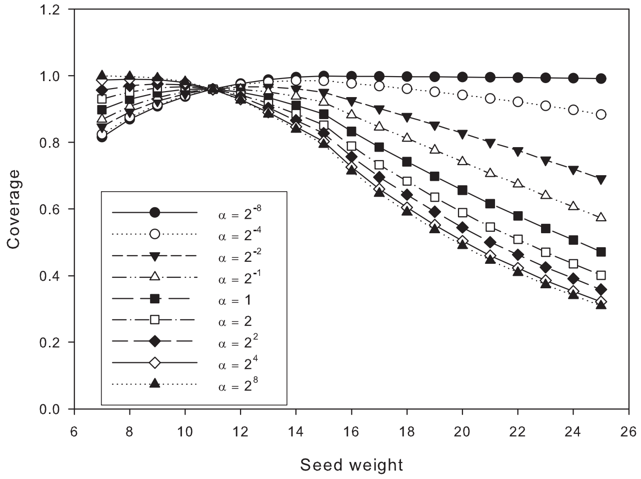

Figure S2: Discriminability according to values of α

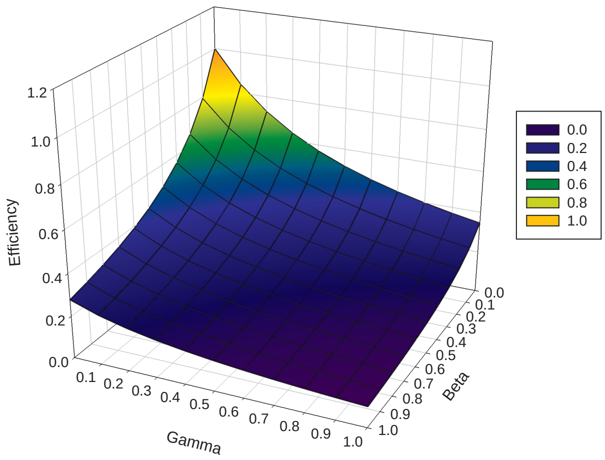

Figure S3: Efficiency according to values of β and γ
